# A qualitative study of women and partners from Lebanon and Quebec regarding an expanded scope of noninvasive prenatal testing

**DOI:** 10.1186/s12884-020-03538-y

**Published:** 2021-01-13

**Authors:** Hazar Haidar, Jessica Le Clerc-Blain, Meredith Vanstone, Anne-Marie Laberge, Gilles Bibeau, Labib Ghulmiyyah, Vardit Ravitsky

**Affiliations:** 1 Institute for Health and Social Policy, McGill University, Montreal, Canada; 2 Medical Genetics, Department of Pediatrics, and Research Center, Centre Hospitalier Universitaire Sainte-Justine, Montréal, Canada; 3 Department of Family Medicine, McMaster University, Hamilton, Canada; 4 Medical Genetics, Department of Pediatrics, and Research Center, Centre Hospitalier Universitaire Sainte-Justine, Montreal, Canada; 5 Department of Pediatrics, Faculty of Medicine, Université de Montréal, Montreal, Canada; 6 Department of Social and Preventive Medicine, École de Santé Publique, Université de Montréal, Montreal, Canada; 7 Department of Anthropology, Faculty of Arts and Sciences, Université de Montréal, Montreal, Canada; 8 Department of Obstetrics and Gynecology, American University of Beirut, Beirut, Lebanon; 9 Bioethics Program, Department of Social and Preventive Medicine, School of Public Health, Université de Montréal, Montreal, Canada

**Keywords:** Noninvasive prenatal testing, Qualitative, Interviews, Lebanon, Quebec, Cultural contexts, Future uses, Paternity, Fetal whole genome sequencing, Sex selection

## Abstract

**Background:**

In the near future, developments in non-invasive prenatal testing (NIPT) may offer couples the opportunity to expand the range of genetic conditions tested with this technology. This possibility raises a host of ethical and social concerns, such as the type of information (medical vs. non-medical information) that couples might be exposed to and how this might complicate their informed decision-making. Currently, only limited research, mainly carried out in western countries, was conducted on women’s and partners’ views regarding the potential expansion of NIPT.

**Methods:**

This study used semi-structured interviews with pregnant women and their partners to explore their views on future potential NIPT applications such as non-medical sex selection and non-medical traits, paternity testing, and NIPT use for fetal whole genome sequencing (FWGS). It was conducted in Lebanon and Quebec, as case studies to explore the impact of cultural differences on these views.

**Results:**

We found no differences and many similarities when comparing the perceptions of participants in both contexts. While couples in both settings disapproved of the use of NIPT for non-medical sex selection and non-medical traits such as physical characteristics, they were near-unanimous about their support for its use for paternity testing in specific cases, such as legal doubts or conflicts related to the identity of the father. Participants were more ambivalent about NIPT for Fetal Whole Genome Sequencing. They supported this use to detect conditions that would express at birth or early childhood, while objecting to testing for adult-onset conditions.

**Conclusions:**

These results can further inform the debate on the future uses of NIPT and future policy related its implementation.

**Supplementary Information:**

The online version contains supplementary material available at 10.1186/s12884-020-03538-y.

## Background

Introduced into clinical practice in 2011, cell-free DNA (cfDNA)-based noninvasive prenatal testing (NIPT) is a screening tool that analyzes cfDNA circulating in maternal blood to test for fetal aneuploidies. NIPT has created a shift in the practice of prenatal screening and diagnosis due to its non-invasive nature (the test does not carry a risk of miscarriage), its ability to be performed as early as 9 weeks of pregnancy, and its reliability in detecting fetal aneuploidies such as trisomy 21 [1, 2]. NIPT is regarded as an advanced prenatal screening method that is superior to existing ones [3, 4] and its results should be confirmed with a diagnostic procedure such as amniocentesis or chorionic villus sampling [5]. Current clinically available uses of NIPT include screening for chromosomal conditions such as trisomy 21, trisomy 13 and trisomy 18, sex chromosome aneuploidies, as well as microduplication and microdeletion syndromes [6]. NIPT has a wide range of potential uses, such as non-medical testing for paternity and fetal sex [7].

The “next frontier” in prenatal testing would be sequencing the entire genome of a fetus through NIPT [8] (p.14173). The proof of concept of such sequencing has already been demonstrated, implying that the test could, in the near future, identify a much wider range of fetal genetic disorders [9, 10]. Fetal Whole Genome Sequencing (FWGS) through NIPT will generate a significant volume of genetic information that will be available prenatally, raising a host of ethical, social and legal concerns. For instance, ethicists, clinicians and the public raised questions regarding the information that should or should not be provided to couples, appropriate ways of communicating results, and the impact of such information on the future autonomy of the child [11, 12]. In light of the developments in NIPT technology, the European Society of Human Genetics (ESHG) and the American Society of Human Genetics (ASHG) issued a joint statement in 2015 recommending a cautious approach to the expansion of prenatal testing to screen for serious congenital and childhood disorders [13].

Previous studies have explored the views, attitudes and experiences of women and their partners regarding the use of NIPT to detect fetal aneuploidies [14–18]. Nevertheless, only scant literature exists on their perceptions and views regarding the evolving applications of NIPT [19–22], and almost all of it consists of quantitative studies conducted in western countries such as the US, the Netherlands and Australia. Consequently, an in-depth understanding of this population’s views regarding a wider range of NIPT application is lacking.

This study thus aims to explore the perceptions and views of women and their partners who have accepted or declined NIPT towards its use for a variety of conditions, including paternity testing, sex determination for non-medical reasons, and the use of NIPT for FWGS. Since cross-cultural differences can have important implications for NIPT decision-making, we chose to conduct semi-structured interviews with pregnant women and their partners in Quebec (the largest province in Canada) and Lebanon. We chose these locations as case studies to investigate whether and how cultural contexts might impact participants’ views about future applications of NIPT. Table 1 represents a summary of the contextual backgrounds for Lebanon and Quebec. For a full description of the cultural contexts, as well as the legal and healthcare systems in both jurisdictions, please refer to our previous publication [23].

**Table 1:** Summary of the contextual background for Quebec and Lebanon

Country	Quebec	Lebanon
**Structure of the healthcare system**	Public; meaning that the provincial government is the principal administrator of healthcare services.	Private and public; meaning that healthcare services are provided through both the public and the private sectors.
**Coverage of prenatal tests**	Prenatal tests are covered by the healthcare system if medically indicated and hence, prescribed by the physician. As for NIPT, it started to be covered in Quebec for women with high-risk pregnancies in July 2020.	Prenatal tests are generally not covered unless one benefits from a public or a private coverage. Regarding NIPT, it is paid for out of pocket.
**The legal status of abortion**	Abortion in Quebec is legal at any time during pregnancy with late term abortions e.i. after 24 weeks, performed in few clinical cases and in restricted number of institutions.	According to the Lebanese penal code, abortion is illegal except to save the mother’s life. However, the law is not enforced, and it is being clinically practiced in a clandestine manner.

## Methods

### Data collection

In both Quebec and Lebanon, potential participants included pregnant women at low and high risk of having a child with trisomy 21, who were identified by recruiters through their medical records.

Women were classified as high risk because of either their maternal age or their medical history with previous pregnancies, or because their MSS result was positive for Down syndrome.

In Montreal (Quebec, Canada), participants were approached by the study coordinator or a nurse following their clinical consultation about prenatal testing at the obstetrics/gynecology and medical genetic clinics at a large university center. In Beirut (Lebanon), participants were recruited by a nurse or a physician at a Women’s Health Center within a Department of Obstetrics and Gynecology at a large Medical Center.

We conducted one-on-one or one-on-two semi-structured interviews with pregnant women or couples (i.e. pregnant women and their male partners). Women chose whether or not they wished to include their partner in the interview. To ensure the feasibility of the study and to have a homogeneous sample in both cultural contexts, only heterosexual couples were recruited. Interviews were audiotaped, transcribed verbatim, and anonymized. In both Montreal and in Beirut, the semi-structured interview guides explored the same topics, including general attitudes regarding NIPT, informed consent for NIPT, NIPT coverage by the health care system and insurance companies, current and future NIPT uses, and social impact of NIPT. The interviews relied on participants’ knowledge of NIPT. However, the question about the use of NIPT for FWGS included a definition of FWGS.

The interview guide is provided as a supplementary file to the manuscript (see Additional file 1).

### Data analysis

We used a thematic approach along with the help of the software package NVivo 11 to analyse our collected data. H.H. and G.B. independently coded the transcripts for interviews in Montreal and another two researchers (H.H. and C.H.) independently coded the Arabic transcripts. Researchers then compared the coded transcripts and discussed discrepancies until they reached consensus. H.H. translated all themes to English, so authors were able to discuss the developing analysis and prepare the manuscript. G.B. validated the French-English translations and C.H. validated the Arabic-English translations. H.H. selected quotes from the interviews to illustrate the findings.

### Participant characteristics

In total, we approached 61 individuals: 11 Quebecois couples, 12 Quebecoise women, 8 Lebanese couples, and 11 Lebanese women. Forty-three individuals accepted to participate in the interviews: 7 Quebecois couples, 8 Quebecoise women, 6 Lebanese couples, and 9 Lebanese women (Table 2). All interviews were conducted by H.H.. In Montreal, we interviewed seven couples (women and their male partners), and eight pregnant women (without their partners) between October 2014 and March 2015. Three interviews were conducted face-to-face and 12 by phone; 14 were conducted in French, and one in English. In Beirut, we interviewed six couples and nine pregnant women between June 2015 and August 2015. We carried out all interviews face to face, with 13 interviews conducted in Arabic and two in English. All participants received a USD30 honorarium for their time; four participants declined the honorarium.

**Table 2:** Participants’ demographics

	Quebec		Lebanon	
	Pregnant women (*N* = 15)	Male partner (***N*** = 7)	Pregnant women (*N* = 15)	Male partner (***N*** = 6)
**Age at delivery (mean = yrs)**	Mean = 35.2	Mean = 34	Mean = 31.3	Mean = 35.3
24–29 years	0	1	6	1
30–33	5	2	4	2
34–39	9	3	5	1
40+	1	1	0	2
**Gravidity**				
First	5		9	
Second	4		3	
Third	4		2	
Fourth	1		1	
Fifth	1		0	
**Prenatal testing history**				
Unknown risk-the couple declined all prenatal tests	0		1	
Low-Risk- opted for traditional screening	5		8	
Low-Risk- opted for NIPT	0		1	
High-Risk- opted for invasive testing (amniocentesis)	9		0	
High-Risk- opted for NIPT	1		1	
High-Risk- Declined further tests including NIPT and amniocentesis	0		4	
**Religiosity**				
Considers self “religious”	11	6	14	6
Christian	11	5	6	3
Muslim	0	1	5	1
Druze	0	0	2	2
Christian + Muslim	0	0	1	0
Does not consider self “religious”	4	1	0	0
Prefers not to answer	0	0	1	0
**Highest level of education completed**				
Primary school	0	0	1	0
High school	1	1	3	1
College	7	1	1	0
University undergraduate	4	3	4	3
University graduate or professional	3	2	6	2

### Research design

In this study we aimed to elicit direct information about future uses of NIPT and to understand how participants view such uses. Qualitative description (QD) methodology enabled us to attain this objective [24]. QD offers straightforward account of events and experiences while providing comprehensive explanation of the said topic as viewed and experienced by the participants in the study. QD research is also culturally-sensitive in that it provides rich information about concerns and issues that are grounded both in environmental and cultural contexts [24, 25]. This attribute is particularly important when working with different cultures [26] to explore the cultural nuances pertaining to the subject of interest, allowing therefore a rich description reflected through the voices of participants and framed by context. We used the QD design to explore how participants in particular contexts (Montreal and Beirut) view different future uses of NIPT. The study received ethics approval from the research ethics committee of the medical centers in which participants were recruited. Written informed consent was obtained from all study participants prior to data collection and all data were anonymized.

## Results

When asked about their views regarding future uses of NIPT, including for non-medical sex-selection, non-medical traits, paternity testing, and FWGS, participants in both settings expressed a variety of opinions and concerns. No striking differences were found between women and their partners. Moreover, remarkable similarities and agreement were found between participants in the two different cultural contexts.

### Non-medical sex-selection and non-medical traits

Quebecois participants were enthusiastic about knowing the sex of the fetus through NIPT. However, when they were asked what they thought about using the test to select the sex of the fetus for non-medical reasons, none of them agreed. Participants mentioned different reasons for thi objection, such as such selection expressing*“playing God”* (*Qc 9 Cp F)* and being*“against nature” (Qc 1 F).* Moreover, they feared that some women or couples would request a pregnancy termination because the fetus is of the unwanted sex without stating the real reason behind their request.

*No. It's just because in my eyes it's playing God when we decide whether or not to keep a child based on sex. I am well aware that there are surely women who are having abortions by citing some other [reason], it might be the same case there. Qc 9 Cp F*

One Quebecois woman noted that she would not mind if NIPT were offered with a restriction to not reveal the sex before the point in pregnancy after which the fetus would be viable outside the womb, to prevent terminations for sex selection:

*If NIPT was offered to me saying we will not divulge the result of the [fetus’] sex before x weeks, like after viability, because we do not want people to select based on it. We do not want the test to be used for selections based on that, it would not bother me, I don’t care. Qc 8 Cp F*

Two Quebecois participants (2/15) did not object to using NIPT to test for cosmetic traits such as physical characteristics - among others, eye and hair colors - *“out of interest and curiosity”*, whereas the majority rejected such use. Some were convinced that one should allow nature to run its course and should reserve such *“surprises”* until the delivery of the child. In addition, while some participants were convinced that in a very advanced future there might be a potential tendency to use NIPT to test for physical traits, they argued that one has to *“accept what God gives you” (Qc 1 F)* and *“should not change the natural” (Qc 1 F)*. Participants also feared a *“slippery-slope”* towards eugenics, which could lead to people starting to test, terminate and *“try again” (Qc 4 Cp H)* in order to have a baby with desired characteristics.

*Oh yes, my child has eyes that are brown, I wanted them blue, oh yes, we go ahead and we start over! In China, what happens is that no one wants a girl. So, with the science, they check quickly, they see that it’s a girl, they terminate. It actually changes the ratio of women to men. And what will happen there? It’s hard to determine all that! Science is good, but there will be abuse on that front. That’s it actually, there will be abuse. They will say; o.k. I want to have a child with blue eyes with long hair. They will terminate, terminate, terminate. That is the not so cool side of this thing. Qc 4 Cp M*

As for Lebanese participants, they were also enthusiastic about knowing the sex of the fetus through NIPT. However, they totally disapproved of its use for non-medical sex selection. They mentioned different rationales, such as*“I accept whatever God gives me” (Lb 1 F)*, *“it’s against religion”(Lb 8 Cp H)*, and *“it is an ethical issue before a religious one” (Lb 10 Cp F)*:

*Of course not. It is related to religion and it’s about culture. Whatever God gives me I will accept it, being healthy is more than just a blessing*. *Be it five girls or ten boys! Lb 9 Cp F*

*No, I already don’t like that because for me it’s against nature, what will happen will happen, you don’t have to choose, it’s weird. And it’s a luxury but I wouldn’t feel comfortable doing that. Lb 7 F*

However, one Lebanese woman stated that if NIPT becomes commercially available people might consider it for such use:

*Personally, I am against it and I wouldn’t do it. However, people might consider it for such purpose if it becomes commercially available. Lb 15 F*

When asked about the use of NIPT for non-medical traits, all other participants rejected such use, except for one Lebanese couple who approved of using it to test for cosmetic traits such as physical characteristics (such as eye and hair colors) out of interest and curiosity:

*There is no problem in using it for this purpose. I am not against it. Lb 10 Cp F*

*Why not. It would be of interest for me. Just to know for example if his hair is like his mother’s or his father’s. Lb 10 Cp M*

Some were convinced that you should allow nature to take its course and you should reserve such “surprises” (*Lb 3 Cp F)* until the delivery of your child. Many other participants argued that you have to accept what God gives you and you should not let humans control such things:

*Again it is something against the concept of creation. It is God who decides such things and I do not like it to be controlled by humans. God sent you this gift and you have to accept it as it is. Lb 12 F*

*No. It would be nice to leave such things as a surprise in order to enjoy it. We will know about all of that sooner or later. Lb 3 Cp F*

### NIPT use for paternity testing

Only one Quebecois participant disapproved the use of NIPT for paternity testing because it could lead to termination of pregnancies and suggested to perform such test only after delivery.

*No, I would not agree to that test either. Again, I think there would be children who would have died today. I think it should be done when the child is born, at that time. Qc 7 Cp F*

However, all other participants, whether in Lebanon or in Quebec, were interested in such use. They argued that even if it might not be of personal interest for them to use NIPT for paternity testing, it would be helpful for other couples in case of doubts or conflicts related to the identity of the father or in case a woman with different partners wants to identify the father. Nevertheless, participants were convinced that such use must be on request only or on a case-by-case basis and that both partners should consent to it.

*If it is to determine paternity, well if there are any doubts, if someone requires it, O.k. But from the outset, I would not do the test from the outset to say oh yes, it’s true that you are the father and the mother. So, provided it’s on request. Qc 9 Cp M*

*Yeah […] but I think not without the exclusive consent of the parents, because otherwise there will be incidental non paternity findings that may destabilize people. It may be an option that is requested by families, but both parents must consent to it. Qc 8 Cp F*

*Yes, I agree if there is a reason behind it. However, it should not be done automatically just to know who the father is, and it should be specific to each case. Lb 6 F*

*If there is any conflict within the couple and they both agreed to do it there is no problem. They both should decide if they want to go for it or not. Lb 10 Cp F*

### NIPT use for fetal whole genome sequencing (FWGS)

Participants were asked if they were interested in using NIPT to know the entire genetic sequence of their fetus. Although different opinions emerged, they were very similar when comparing Lebanese and Quebecois participants’ views.

Some Quebecois participants completely disapproved of using NIPT for FWGS, because they thought it will create anxiety for both parents and their future child. Parents will be distressed because of a risk that their child will develop a certain condition, leading sometimes to a decision of pregnancy termination based on a probability. Furthermore, they thought parents will be watching over their child, over-interpreting any change or sign and overprotecting him/her, while waiting for something to happen, which will in turn negatively impact the child’s life.

*Imagine that you know that your child will develop a certain disease when he gets 15 years old. All these fifteen years of his life will have an impact of what will be happening. So, he will not have lived, he will be watched over for any change in anticipation for what might happen. Qc 14 F*

This is not something I would like to know because I run the risk of being anxious in advance when we are in the future. And I would not abort a child who is at risk of having troubles let’s say in the heart or […] It’s clearly that, it’s just that I might worry about risks. If the test said, I don’t know, at 10 years old he’ll develop such a disease because of such a genome, that’s another story! Here we talk about risks, statistics, I would rather not know it. Qc 9 Cp F

Several participants showed ambivalence: while they were interested in NIPT for FWGS to detect diseases that might develop at birth or during childhood in order to *“prevent or treat if possible”* those conditions or ‘to prepare’ for the child’s birth, they disapproved of it for having information related to adulthood. The reason behind it is that once he becomes an adult, the child should have a choice about whether or not he wants to know such information:

*Me, for me I would do it. My child, I would like to know things that would be relevant in childhood. Let’s say if he’ll develop an infantile cancer or a childhood disease, or something like that, I would like to know. But things like at age 30, at age 40, at that time they’ll make the choice, at age 18 to be tested themselves.* […] *I want them to choose to know for themselves. Qc 8 Cp F*

Only one Quebecois couple approved of using NIPT for FWGS: the woman was in favor of its use to detect neonatal and childhood conditions, while her partner argued that he would also be interested in knowing his future child’s risk of developing diseases during adulthood. He stated that he would not necessarily disclose this information to his child until he becomes an adult and depending on the condition he might develop, as exemplified by the following quote:

*Personally, I would still prefer to know if he’ll develop Alzheimer’s or other diseases at 30, 40 years, even if nothing can be done. If it would be information about my child, I would prefer to know it too. I would not necessarily tell him right away, I would wait for him to grow up, depending on the condition. But personally yes, I would like to know it. Qc 8 Cp M*

Others agreed and were interested in NIPT to sequence their fetus and know about future risks, in order to make a decision regarding whether to terminate or continue the pregnancy:

*Yes, without hesitation, yes. Because if you carry a child and you get to know what illness the child will develop in the long run, we might terminate the pregnancy, or we accept that the child lives with these things.*

Most participants agreed that if it would be possible to test for more conditions or diseases, those that are life-threatening or related to the quality of life of the future child should be targeted. Moreover, they emphasized the need to “*draw the line somewhere between those severe diseases that are incompatible with life and physical traits such as blue eyes” (Qc 12 F)*.

We noticed Quebecois participants and Lebanese participants shared similar views. For instance, some Lebanese participants completely disapproved of using NIPT for FWGS because they considered it to create anxiety for both parents and the future child, or based on the notion that what happens in the future should remain only in “*God’s hands*” and be “*God’s will*” (*Lb 3 Cp F)*.

*No, I am against it because it’s all about God’s will. Even if the fetus has a problem, in the end I think that by terminating a pregnancy we will be killing a soul. This is why I am against it. I accept God’s will because no one can predict what will happen tomorrow. Lb 3 Cp F*

*And other than that, we might know that our child will develop cancer in 15 years. It is bad for him even bad for us to know such information because we will be anxious and waiting for the disease to develop, so let it be as it is. Lb 3 Cp M*

Several Lebanese participants approved of the use of NIPT for FWGS to detect conditions that might develop at birth or during childhood in order to prevent or treat them or to manage their pregnancy (prepare for the child’s birth or terminate the pregnancy), nevertheless they disapproved of its use to obtain information related to conditions with onset in adulthood.

*Honestly, I would be interested if it will give me results related to conditions that develop at birth. Because for instance, if, God forbid, my child will have any problem when he is born, I might be able to help him. But I will not go for it to know about the risk of conditions that might or might not develop later on because I do not want to be anxious throughout my whole life, thinking and waiting for the disease to appear at a certain age. Lb 11 F*

Moreover, some stated that they would perform NIPT for FWGS out of curiosity while deciding not to do anything about the pregnancy:

*This is controversial because you never know what will happen. Maybe he will develop Alzheimer’s but after many years there will be a cure for Alzheimer’s. However, for curiosity’s sake, I will certainly go for it. But will I be doing anything about my pregnancy? No, I will not. Lb 9 Cp F*

*If I can know what is waiting for me, and what is the baby’s risk to develop a disease at birth or even later on during his life, I would definitely go for it. Maybe we can prevent or treat and if not, at least I will know what to expect and I will be prepared. Lb 12 F*

## Discussion

This study provides an insight into the opinions and views of participants regarding potential future uses of NIPT from two different cultural contexts: Quebec and Lebanon. The most interesting finding is the striking similarities between the views of participants from both settings. We expected to find differences because of the specific cultural and social backgrounds characterizing each context. We also expected differences due to findings we published previously, based on our analysis of different themes, showing how these backgrounds shape and influence pregnant women and couples’ decision-making surrounding NIPT [23].

Most participants from both settings were enthusiastic about knowing the sex of the fetus through NIPT. However, they were unanimously against considering it for non-medical sex selection. Objections to the use of NIPT for sex selection were also reported by other studies, where diverse stakeholders including the public [27], pregnant women and women [18, 19, 21], and their partners [21] disapproved of NIPT for sex selection. Interestingly, only Quebecois participant feared that some individuals would terminate a pregnancy based on the sex of the fetus. This concern might be tied to the debate surrounding termination of pregnancy, which might in turn be considered within local cultural contexts and policies that shape reproductive decision making.

NIPT may facilitate couples and pregnant women’s choice by offering them information regarding the fetus’s health and hence, allowing them either to prepare for the birth of a child with a certain genetic condition or to consider the option of pregnancy termination.

It is worthy to note that our study considers future uses of NIPT that may be medical or not (such as non-medical sex selection). The concerns expressed by Canadian participants regarding non-medical sex selection through selective termination, need to be understood on the backdrop of the decriminalisation of abortion in Canada and its availability, in principle, throughout the pregnancy [28]. In Canada, women can theoretically (notwithstanding local logistical barriers) access abortion for any reason and their reproductive rights are protected.

This is clearly not the case in Lebanon, where reproductive rights are more restrained by law, since termination of pregnancy is illegal, except to save the mother’s life [23, 29]. On a clinical level, abortion is performed in a clandestine manner and might be accessible for a certain group of women who are able to afford paying for it [29]. Expanding the use of NIPT will therefore have much less influence on Lebanese women’s and couples’ choices surrounding pregnancy management.

Expanding the use of NIPT use for non-medical traits, such as eye colour, was rejected by the vast majority of participants from both contexts. Rationales put forward by participants were that doing so was against God’s will and/or against nature’s course. These justifications have been raised and explored in other studies. For instance, religion and accepting what God gives you was a pronounced theme in a study that surveyed and interviewed Latina women deciding whether to accept or reject NIPT [30]. In another study, performed by van Schendel et al., participants stated that “you should let nature run its course” when they were interviewed about widening the scope of NIPT to include an extended range of genetic disorders, as well as its use for non-medical reasons [21].

Invoking God and God’s will to discuss NIPT use for non-medical reasons by Lebanese participants reflects the social and cultural nature of the Lebanese context. In Lebanon, religion is integrated in state affairs – “personal matters including inheritance, marriage, divorce, custody, and support are dealt with in religious courts” [31] (p.2), and religious leaders are consulted “whenever a new law is to be proposed” [31], especially when it touches areas such as end-of-life and abortion. Hence, religion plays an important role, especially when it comes to decisions related to procreation and family. For instance, in our previous paper, religion was shown to be one of the main factors in Lebanese women’s and partners’ decision-making on whether or not to accept NIPT [23].

However, it was a surprising finding that religious reasons were brought forward by Quebecois participants given that religion is not embedded in everyday or political life especially when compared to Lebanon. In Quebec, since 1960, the State and the Church have been separated, with no involvement of the Catholic church in state affairs [32]. One possible explanation of Quebecois participants invoking God could be that 17/22 (77.2%) of our study participants considered themselves as religious, more specifically Christian. This is a similar proportion to the general Quebec population who identify as Christian (82.2%) [33]. Our findings show that in the context of decisions related to pregnancy, participants were influenced by their religious values and inspired by them to justify their objection to the use of NIPT for non-medical reasons. It is difficult to ascertain whether there was a self-selection bias in our study, i.e. whether Quebecois participants who have more religious backgrounds were more interested in participating in our study.

The majority of participants from Lebanon and Quebec were favourable towards the use of NIPT for paternity testing in specific cases, such as the existence of doubts or conflicts with regards to the identity of the father. This finding does not align with the one in Farrimond & Kelly’s study, where participants mostly did not support paternity testing through NIPT [27] out of fear of potentially increasing terminations for non-medical reasons.

Participants from both countries also shared similar views concerning the use of NIPT for FWGS. Contrary to the other uses explored, participants were more ambivalent when it came to this use. They were interested in FWGS for diseases that develop shortly after birth or during childhood and that are preventable or treatable. Our findings resonate with those from other studies showing that participants were favorable towards testing for childhood-onset conditions, whether or not performed through FWGS [21, 34–36].

However, our participants disapproved of receiving information about adult-onset conditions. Attitudes towards testing adult-onset conditions in the literature are more varied compared to childhood-onset conditions. In the Kalynchuck et al. study (2015), although the acceptability of testing for adult-onset conditions was not as strong as for childhood-onset conditions, 76% of pregnant women and their partners going through first-trimester screening sated they would want to receive fetal whole-exome sequencing (FWES) results for treatable adult-onset conditions, and 74.3% for untreatable adult-onset conditions. Similar results were reported in Sullivan et al.’s study (2019), where 81.2% of pregnant women (≥ 8 weeks) receiving prenatal care stated they would want to receive, following FWGS through NIPT, information about common treatable conditions, 76.3% for serious treatable adult-conditions, 68.9% for fatal non-treatable adult-onset conditions, and 65.5% for common untreatable conditions.

Conversely, participants from other studies were not as interested in testing for adult-onset conditions. In Bowman-Smart et al. (2019), 37.6% of pregnant women who had already undergone NIPT in the past supported the availability of NIPT for non-preventable adult-onset conditions, and 45.0% for preventable adult-onset conditions [18]. Moreover, van Schendel et al. (2014) report that 29% of pregnant women recruited through a Dutch pregnancy website believed that NIPT testing for severe late-onset conditions should be accessible [21]. Our participants considered the severity of the condition (severe and life-threatening diseases), its timing (neonatal, childhood and adulthood), and the quality of life of the future child, as important factors for the acceptability of testing with FWGS, similarly to what has been reported by participants in the study by van Schendel et al. (2014). Further, noteworthy that a study performed by Poulton et al. showed that testing for adult-onset conditions seems to be more acceptable when it is performed through preimplantation genetic diagnosis than through prenatal testing [37]. This can be explained by preimplantation diagnosis avoiding the thorny ethical issues associated with prenatal testing, such as the possible decision to terminate an affected pregnancy.

In sum, our participants seemed to accept NIPT uses for detecting diseases in order to treat or prevent them if possible. However, whenever the discussion stepped into the domain of non-medical uses, they referred to God, religion and nature as justifications for their objections. One possible explanation of this finding could be that referring to God and nature reflects participants’ fear of over control and treating children like products.

Some Quebecois participants spontaneously mentioned that it was the child’s right not to know about adult-onset conditions. This is in line with the Canadian Paediatric Society’s position statement on testing of minors, which states that “for genetic conditions that will not present until adulthood (susceptibility or predictive testing), testing should be deferred until the child is competent to decide whether they want the information” [38] (p.45), as well as recommendations of other professional societies worldwide [39]. This is based on the premise that testing minors for adult-onset conditions hinders their right to an open future, a right based on the respect of the child’s autonomy and privacy. Making decisions for the child when there are no immediate concerns narrows the child’s future options, and hinders his/her right not to know [40] p.23 [40, 41] (Borry et al., 2014, p.20).

However, the child’s right not to know was not addressed by Lebanese participants. This might be explained by the social and cultural contexts in place, where children have little to no autonomy over their decisions, including medical decisions [42], and parents are considered to be the principal decision-makers for the child.

### Strengths and limitations

The comparative nature of our study provides insight into non-Western views, perspectives, and attitudes towards future uses of NIPT, which, to our knowledge, is not yet explored in the literature. In addition, comparative studies allow to shed light on differences, but also similarities between populations. While cross-cultural differences had important implications for NIPT decision-making [23], in this case of expanded use of NIPT, participants shared similar views, irrespective of these cultural differences. This is an unexpected and illuminating finding. Our findings highlight the opportunity for further research to examine a wider range of potential future applications of NIPT in additional populations such as people with disabilities, healthcare professionals, and policy makers.

The description of FWGS provided to participants had been simplified to make it easier to understand. We did not offer a detailed description of conditions that might be detected. For instance, we did not differentiate between preventable and non-preventable adult-onset conditions, which might have affected participants’ responses. Considering that some of the future uses explored in our study were not feasible or not offered at the time the interviews were conducted, participants had to reflect on hypothetical scenarios. It is possible that their reasoning would have been different if they had to make actual decisions.

Further, we noted that in our study, one Lebanese and two Quebecoise women chose NIPT testing. This might be explained by the fact that at the time of the interviews, the cost of NIPT was a barrier to access [23]. Notwithstanding, the low number of adopters of NIPT might have influenced participants’ attitudes towards the potential future uses of NIPT, including FWGS, paternity testing, and physical traits.

In addition, our study participants did not include women with a confirmed diagnosis of aneuploidy. The views of women post-diagnosis may be different, as the diagnosis of their fetus may influence their perceptions regarding the potential future uses of NIPT.

## Conclusion

NIPT is a fast-moving technology, and its uses have expanded since its first introduction. With the introduction of FWGS, its ability to detect a growing number of genetic conditions will expand. The findings of this study suggest that women and their partners, in Quebec and in Lebanon, support the use of NIPT for several traits and conditions that might be available in the future. However, participants still raise concerns about non-medical uses of the test. Moving forward and with future NIPT applications coming closer, it is important to explore users’ views and the acceptability of this potential expansion prior to its implementation, to promote evidence-informed, ethically sound, and culturally sensitive policy decisions.

## Supplementary Information

**Additional file 1.** Interview guide for pregnant women and couples in Lebanon and Quebec. Interview guide used to collect data from pregnant women and couples in Lebanon and in Quebec.

## Data Availability

The qualitative interview data analyzed during the current study are not publicly available because they might potentially include identifying information that could compromise research participant privacy and consent. Sections of anonymized data are available from the corresponding author on reasonable request.

## Abbreviations

AUBMC: American University of Beirut Medical Center
CHUSJ: Centre hospitalier universitaire Sainte-Justine
ESHG: European Society of Human Genetics
ASHG: American Society of Human Genetics
Cp: Couple
CVS: Chorionic villus sampling
F: Female
FWGS: Fetal whole genome sequencing
Lb: Lebanese
M: Male
NIPT: Noninvasive prenatal testing
Qc: Quebecois
