# Supplementary material for: A qualitative study of women and partners from Lebanon and Quebec regarding an expanded scope of noninvasive prenatal testing
Source: BMC Pregnancy Childbirth. 2021 Jan 13;21:54. doi: 10.1186/s12884-020-03538-y (PMC7805222; doi:10.1186/s12884-020-03538-y)
Supplement: Supplementary file 1 — Additional file 1. Interview guide for pregnant women and couples in Lebanon and Quebec. Interview guide used to collect data from pregnant women and couples in Lebanon and in Quebec. [file 12884_2020_3538_MOESM1_ESM.docx]

**NIPT and Informed Consent**

1- Before today did you hear about NIPT? If yes, how?

2- What did you think about NIPT when you first heard about it? Were the infos that you have received sufficient?

3- In your opinion, what are the information regarding NIPT a woman [her partner] should receive in order to make an informed decision regarding the test?

4- In your opinion, who should provide this information?

5- Beyond the information provided by HCP about NIPT, how else would you like to be informed about it (brochures, video, websites, group meetings, other)? Explain.

6- In your opinion, when should NIPT be performed: on the same day at which it is offered or on a different day? Why?

7- After counseling, how do you think should the consent regarding NIPT be provided (verbally or in writing?) And why?

8- What would be the most appropriate way to receive the results (positive or negative) of NIPT?

9- In your opinion, should NIPT be offered directly to the consumer or only through a healthcare professional? Why?

**Decision-making regarding NIPT**

10- (Did you consider the NIPT?) What is/ are the features of NIPT that have the most influence on your decision-making? Explain.

11- what did u like about the test? What are the factors that can influence your decision to take the test or to decline it? How?

12- If your partner disagreed with your decision to test or to refuse NIPT, would that influence your decision? How? [for partners: how would you deal with situations in which you and your partner disagree regarding the test?]

**Current and future uses of NIPT**

13- Have you done (or heard about) any other tests in this pregnancy? Which ones? How did NIPT compare to those tests?

14- What do you think about the use of NIPT for:

- Other than trisomies 21, 13 and 18, according to you what other conditions should be detected by NIPT?

- Sex selection for non-medical reasons?

- Non-medical traits

- Paternity testing

15- In the future, technological advance might allow sequencing the entire genome of the fetus at a reasonable cost. This might give us information about the child future risk to develop certain conditions at birth, during childhood or even during adulthood. Would you be interested in NIPT in order to get the entire genetic sequence of your future child? Why?

**Social impact of NIPT**

16- If NIPT became part of routine tests offered during pregnancy, in your opinion, what impact would that have on society? (The impact on people with disabilities, research and treatment of certain diseases, etc.?)

19- In your opinion, should NIPT be covered by the HCS?

- For which category of women (all women, high-risk only?)?

- For which conditions?

17- If NIPT were to be covered by the healthcare system or insurance companies:

- How would you feel about that?

- Would you feel that women would ‘have to take the test?

18- Currently, NIPT is not covered. Would you be ready to pay for it out-of-pocket? If yes how much? What percentage of your salary would you be willing to spend on this test?

19- Now I just have a few demographic questions before we close:

- At the beginning of your pregnancy, was your main pregnancy caregiver a family doctor, midwife, or obstetrician?

- Before this pregnancy, have you done any other kind of prenatal testing or screening?

- How old are you?

- Do you have any other children?

- What's the highest level of education you have?

- Do you consider yourself religious? If so, what religion do you belong to?
